# Development and validation of a simple-to-use nomogram for self-screening the risk of dyslipidemia

**DOI:** 10.1038/s41598-023-36281-3

**Published:** 2023-06-06

**Authors:** Jinyan Lan, Xueqing Zhou, Qian Huang, Li Zhao, Penghua Li, Maomao Xi, Meng Luo, Qiong Wu, Lixu Tang

**Affiliations:** 1 Martial Arts Academy, Wuhan Sports University, No. 461 Luoyu Rd., Hongshan District, Wuhan, 430079 Hubei China; 2 Physical Examination Center, Renmin Hospital of Wuhan University, Wuhan, China; 3 Hubei Institute of Sport Science, Wuhan, China; 4 Hubei Provincial Hospital of Integrated Traditional Chinese and Western Medicine, Wuhan, China; 5 Tongren Hospital of Wuhan University (Wuhan Third Hospital), Wuhan, China; 6 Lanzhou University Second Hospital, Lanzhou, China

**Keywords:** Cardiovascular diseases, Dyslipidaemias

## Abstract

This study aimed to help healthy adults achieve self-screening by analyzing the quantitative relationship between body composition index measurements (BMI, waist-to-hip ratio, etc.) and dyslipidemia and establishing a logical risk prediction model for dyslipidemia. We performed a cross-sectional study and collected relevant data from 1115 adults between November 2019 and August 2020. The least absolute shrinkage selection operator (LASSO) regression analysis was performed to select the best predictor variables, and multivariate logistic regression analysis was used to construct the prediction model. In this study, a graphic tool including 10 predictor variables (a "nomogram," see the precise definition in the text) was constructed to predict the risk of dyslipidemia in healthy adults. A calibration diagram, receiver operating characteristic (ROC) curve, and decision curve analysis (DCA) were used to verify the model’s utility. Our proposed dyslipidemia nomogram showed good discriminative ability with a C-index of 0.737 (95% confidence interval, 0.70–0.773). In the internal validation, a high C-index value of 0.718 was achieved. DCA showed a dyslipidemia threshold probability of 2–45%, proving the value of the nomogram for clinical application for dyslipidemia. This nomogram may be useful for self-screening the risk of dyslipidemia in healthy adults.

## Introduction

With rapid economic development, secondary lifestyles and the intake of a high-fat diet have increased dramatically, leading to the worldwide prevalence of overweight and obese individuals^1^. Recent statistics show that the incidence of overweight and obese individuals continues to rise globally, with more than 2 billion individuals being overweight accounting for approximately 30% of the world’s population^2,3^. Dyslipidemia, characterized by hypercholesterolemia, hypertriglyceridemia, high low-density lipoprotein cholesterol (LDL-C), and/or low high-density lipoprotein cholesterol (HDL-C), is a major risk factor for cardiovascular disease and obesity^4,5^. Several studies have shown that early prevention and management of dyslipidemia are effective in the primary prevention of cardiovascular events and obesity^6,7^, thus providing a considerable opportunity to reduce the disease burden, having great social value. Many studies report that body mass index (BMI), hip circumference (HC), and waist circumference (WC) as surrogate measures of obesity, correlate positively with dyslipidemia^8–10^. However, the quantitative relationships between these body composition indices measurements and dyslipidemia are not fully understood.

The logistic regression model is a widely used approach to identify risk factors for certain diseases. Based on logistic regression models, a visualization method, namely the nomogram, which graphically and simply represents the numerical relationship between the probability of disease and risk factors without a complex formula, has been developed^11,12^. A nomogram is a novel risk prediction model combining multiple indicators rather than univariate analysis and subsequent multivariate logistic analysis; it is important for screening and clinical practice. The application of the nomogram prediction model can accurately screen relevant variables and indicators and determine the most appropriate risk factors. In this study, we developed a nomogram to illustrate the quantitative relationships between body composition indices measurements and dyslipidemia using least absolute shrinkage and selection operator (LASSO) analysis, an appropriate tool for selecting more favorable variables by re-weighting the LASSO penalty for each variable. Based on this nomogram, healthy adults could self-screen for the risk of dyslipidemia, and dyslipidemia patients could design an exercise plan to maintain a healthy body size and shape to reduce the risk of dyslipidemia.

## Methods

### Study population

This cross-sectional study was approved by the Institutional Review Board (IRB) of Wuhan Sports University and conducted following the principles outlined in the latest version of the Declaration of Helsinki. It was conducted at the Hubei Institute of Sport Science from November 18, 2019, to August 11, 2020, and aimed to observe the effect of body composition index measurements on dyslipidemia. A total of 1115 volunteered to participate and signed the written informed consent letter. From among the 1115 participants, we excluded those who (1) were diagnosed with dyslipidemia (because both exposure and outcome are measured simultaneously in cross-sectional studies, the outcome of interest in this study is undiagnosed dyslipidemia), cardiovascular diseases (including hypertension, myocardial infarction, coronary artery disease, heart failure, peripheral artery disease, or stroke), diabetes, abnormal liver function, abnormal renal function, abnormal thyroid function, malignancy, and/or active inflammatory diseases (n = 15); (2) had no data on their general characteristics (n = 59), including age, marital status, educational level, type of occupation, smoking status, frequency of physical activity, previous medical history, and/or annual income; (3) had no data on their body composition measurements (n = 26). The final study sample included 1015 participants of which 495 were males and 520 were females, aged 19–68 years.

### General characteristics

The general characteristics were obtained through a questionnaire that was completed by each participant including the following information: name, sex, age, marital status, educational level, type of occupation, smoking behavior, drinking status, frequency of physical activity, previous medical history, and annual income. According to the latest regulations of the United Nations World Health Organization (WHO) on the classification criteria of age groups, participants were grouped into youth (age < 44 years), middle-aged (44 years ≤ age ≤ 60 years), and elderly (age > 60 years) categories^13^. Marital status was classified into two groups, namely living alone (including single, divorced, separated, or widow/widower) and living as a couple (including married, cohabitant, and other relationships). The educational level was divided into two groups, namely those with tertiary education (university education or higher) and those without. The type of occupation was divided into manual and non-manual by self-reporting. Smoking behavior was classified as never smoking, formerly smoking (having not smoked for more than 6 months), and currently smoking (having smoked at least one cigarette within the past 6 months). Alcohol intake was also classified into two groups, i.e., consuming alcohol presently or not (drinking alcohol in the past or never drinking). Physical activity was classified into the following three categories: none, irregular (≤ 2 episodes/week), and regular (≥ 3 episodes/week). Annual income was categorized as < 100,000 RMB and ≥ 100,000 RMB groups.

### Body composition indices measurements

Height, weight, hip circumference (HC), waist circumference (WC), weight, fat mass, and body fat percentage were measured for participants when they were dressed in light clothing without shoes by trained staff following the standard procedures as body composition indices. The height was measured to the nearest 0.1 cm by using a stadiometer (Seca). WC and HC were measured with a nonelastic measuring tape to the nearest 0.1 cm. Weight and body fat percentages were measured using a direct segmental multi-frequency bioelectrical impedance analyzer (In Body 770). All measurements were repeated thrice, and the mean value was used in this study. We also calculated body mass index (BMI) as weight (kg) divided by squared height (m); waist-to-hip ratio (WHR) as WC (cm) divided by HC (cm); waist-to-height ratio (WHtR) as WC (cm) divided by height (cm), and hip-to-height ratio (HHtR) as HC (cm) divided by height (cm). The WHR, WHtR, HHtR, and body fat percentages were categorized based on the best cut-off points obtained from the receiver operating characteristic (ROC) curve analysis. BMI was classified as follows: < 18.5, 18.5–24.9, 25.0–29.9, and ≥ 30 using the WHO international standards^14^.

### Serum lipid measures and the definition of dyslipidemia

After fasting overnight, the peripheral blood samples of participants were collected to measure the following variables: total cholesterol (TC), triglycerides (TG), low-density lipoprotein cholesterol (LDL-C), and high-density lipoprotein cholesterol (HDL-C). All measurements were recorded in the Hubei Provincial Hospital of Integrated Chinese and Western Medicine using the same and standard procedures. According to the 2016 Chinese Guidelines for the Management of Dyslipidemia in Adults^15^, dyslipidemia was defined as having TC ≥ 6.2 mmol/L, TG ≥ 2.3 mmol/L, LDL-C ≥ 4.1 mmol/L, and/or HDL-C ≤ 1.0 mmol/L.

### Statistical analysis

All data analyses were performed with R software (version 4.0.3; https://www.R-project.org). A univariate logistic regression analysis was performed to compare the differences between the non-dyslipidemia and dyslipidemia groups and calculate odds ratios (OR) and p-values. The LASSO method was used to select variables, before building the predictive model to pick out the optimal variables and eliminate redundant ones^16^. Then, based on the selected variables from the LASSO regression model, multivariable logistic regression analysis was performed and a visual nomogram was constructed as a predictive model.

For assessing the nomogram's accuracy, we used two methods. First, C-index was measured, and internal validation was performed by the bootstrapping technique (1000 bootstraps) to quantify the discrimination of our proposed dyslipidemia nomogram^17^. The R package (Hmisc package) released by Harrell for calculating the C-index was used. Second, the clinical decision curve analysis (DCA) was performed to evaluate the predictive effect of the nomogram and calculate its net benefit^18^. Unless otherwise stated, p-values < 0.05 were considered significant.

## Results

### Participant characteristics

Among the 1015 participants, 495 were males and 520 were females, and their median age was 41 (min = 19; max = 68) years. The proportion of first recorded dyslipidemia cases was 21.48%, with statistically significant differences by sex (p = 0.001), marital status (p = 0.021 < 0.05), educational level (p = 0.025 < 0.05), type of occupation (p = 0.015 < 0.05), smoking behavior (p = 0.001 < 0.05), drinking status (p < 0.001), WHR (p < 0.001), WHtR (p < 0.001), HHtR (p = 0.006 < 0.01), BMI (p < 0.001), and body fat percentage (p < 0.001). The participant characteristics are shown in Table 1.

**Table 1:** Differences between non-dyslipidemia and dyslipidemia groups.

	Total (n = 1015)	n (%)		OR (95% CI)^a^	*p-*value^a^
		Non-dyslipidemia (n = 797)	Dyslipidemia (n = 218)		
Sex					
Male	495 (48.77)	342 (42.91)	153 (70.18)		
Female	520 (51.23)	455 (57.09)	65 (29.82)	0.319 (0.231–0.441)	< 0.001
Age, years					
< 44	668 (65.81)	526 (66.00)	142 (65.14)		
44–60	252 (24.83)	194 (24.34)	58 (26.61)	0.936 (0.643–1.442)	0.085
> 60	95 (9.36)	77 (9.66)	18 (8.25)	0.867 (0.480–1.565)	0.105
Marital status					
Living alone	206 (20.30)	174 (21.83)	32 (14.68)		
Living in couple	809 (79.70)	623 (78.17)	186 (85.32)	1.623 (1.076–2.449)	0.021
Tertiary education					
No	344 (33.89)	284 (35.63)	60 (27.52)		
Yes	671 (66.11)	513 (64.37)	158 (72.48)	1.458 (1.047–2.029)	0.025
Type of occupation					
Non-manual	452 (44.53)	339 (42.53)	113 (51.83)		
Manual	563 (55.47)	458 (57.47)	105 (48.17)	0.688 (0.509–0.929)	0.015
Smoking behaviour					0.001
Never smoking	831 (81.87)	672 (84.32)	159 (72.94)		
Former smoking	21 (2.07)	14 (1.76)	7 (3.21)	2.113 (0.839–5.322)	0.112
Current smoking	163 (16.06)	111 (13.93)	52 (23.85)	1.980 (1.365–2.872)	< 0.001
Drinking status					
Not current drinking	833 (82.07)	672 (84.32)	161 (73.85)		
Current drinking	182 (17.93)	125 (15.68)	57 (26.15)	1.903 (1.331–2.721)	< 0.001
Physical exercise					
None	199 (19.61)	142 (17.82)	43 (19.72)		
≤ 2 episodes/week	663 (65.32)	454 (56.96)	101 (46.33)	1.269 (0.904–1.781)	0.169
≥ 3 episodes/week	153 (15.07)	201 (25.22)	74 (33.95)	2.131 (0.843–5.377)	0.121
Annual income, RMB					
< 100,000	690 (67.98)	550 (69.01)	140 (64.22)		
≥ 100,000	325 (32.02)	247 (30.99)	78 (35.78)	1.241 (0.905–1.700)	0.180
WHR					
< 0.85	300 (29.56)	269 (33.75)	31 (14.22)		
≥ 0.85	715 (70.44)	528 (66.25)	187 (85.78)	3.073 (2.045–4.619)	< 0.001
WHtR					
< 0.52	571 (56.26)	485 (60.85)	86 (39.45)		
≥ 0.52	444 (43.74)	312 (39.15)	132 (60.55)	2.386 (1.756–3.242)	< 0.001
HHtR					
< 0.56	250 (24.63)	212 (26.60)	38 (17.43)		
≥ 0.56	765 (75.37)	585 (73.40)	180 (82.57)	1.717 (1.170–2.519)	0.006
BMI, kg/m^2^					< 0.001
< 18.5	55 (5.42)	52 (6.52)	3 (1.38)		
18.5–24.9	629 (61.97)	531 (66.62)	98 (44.95)	3.199 (0.979–10.448)	0.054
25.0–29.9	288 (28.37)	189 (23.71)	99 (45.41)	9.079 (2.765–29.812	< 0.001
≥ 30	43 (4.24)	25 (3.14)	18 (8.26)	12.480 (3.361–46.347)	< 0.001
Body fat percentage					
< 18%	180 (17.73)	159 (19.95)	21 (9.63)		
≥ 18%	835 (82.27)	638 (80.05)	197 (90.37)	2.338 (1.443–3.787)	0.001

*WHR* Waist-to-Hip Ratio, *WHtR* Waist-to-Height Ratio, *HHtR* Hip-to-Height Ratio, *BMI* Body mass index.

^a^Taking the first as the reference, OR (95% CI) and *p*-values were calculated by univariate logistic regression analysis.

### Feature selection

**Figure 1:**
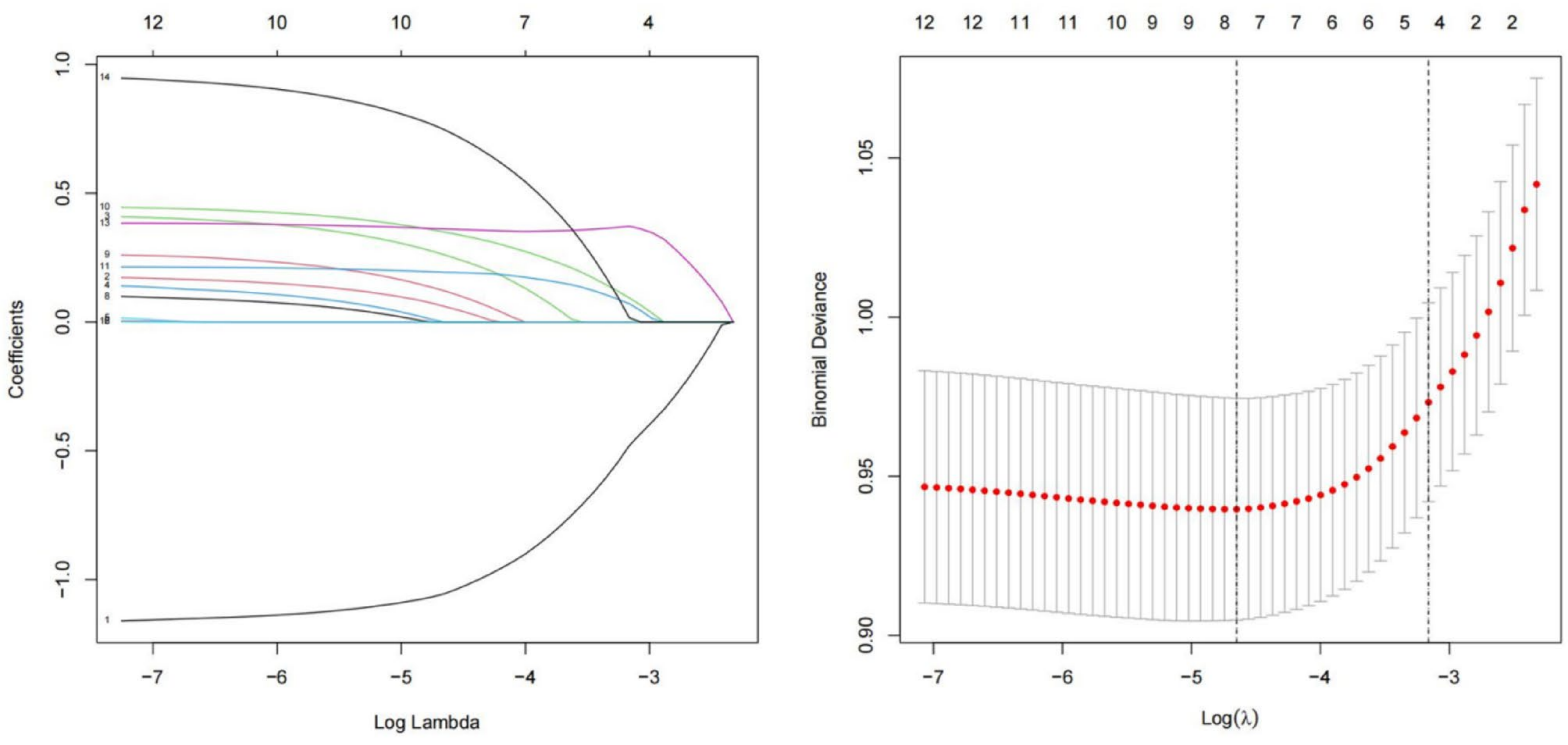
Variable selection using the LASSO binary logistic regression model. (**A**) LASSO coefficients of 14 variables. The optimal penalization coefficient (lambda) was identified in the LASSO model where optimal lambda resulted in ten features with nonzero coefficients. (**B**) The partial likelihood deviance (binomial deviance) curve was plotted versus log(lambda). The left vertical line is dotted at the minimum criteria of lambda, and the right vertical line is dotted at one standard error of the minimum lambda. *LASSO* least absolute shrinkage and selection operator.

Of all the potential variables, 10, including sex, age, marital status, educational level, physical exercise, annual income, WHR, WHtR, BMI, and body fat percentage, were retained in the LASSO binary logistic regression model at the minimum criteria of lambda (Fig. 1).

### Construction of the prediction model

**Figure 2:**
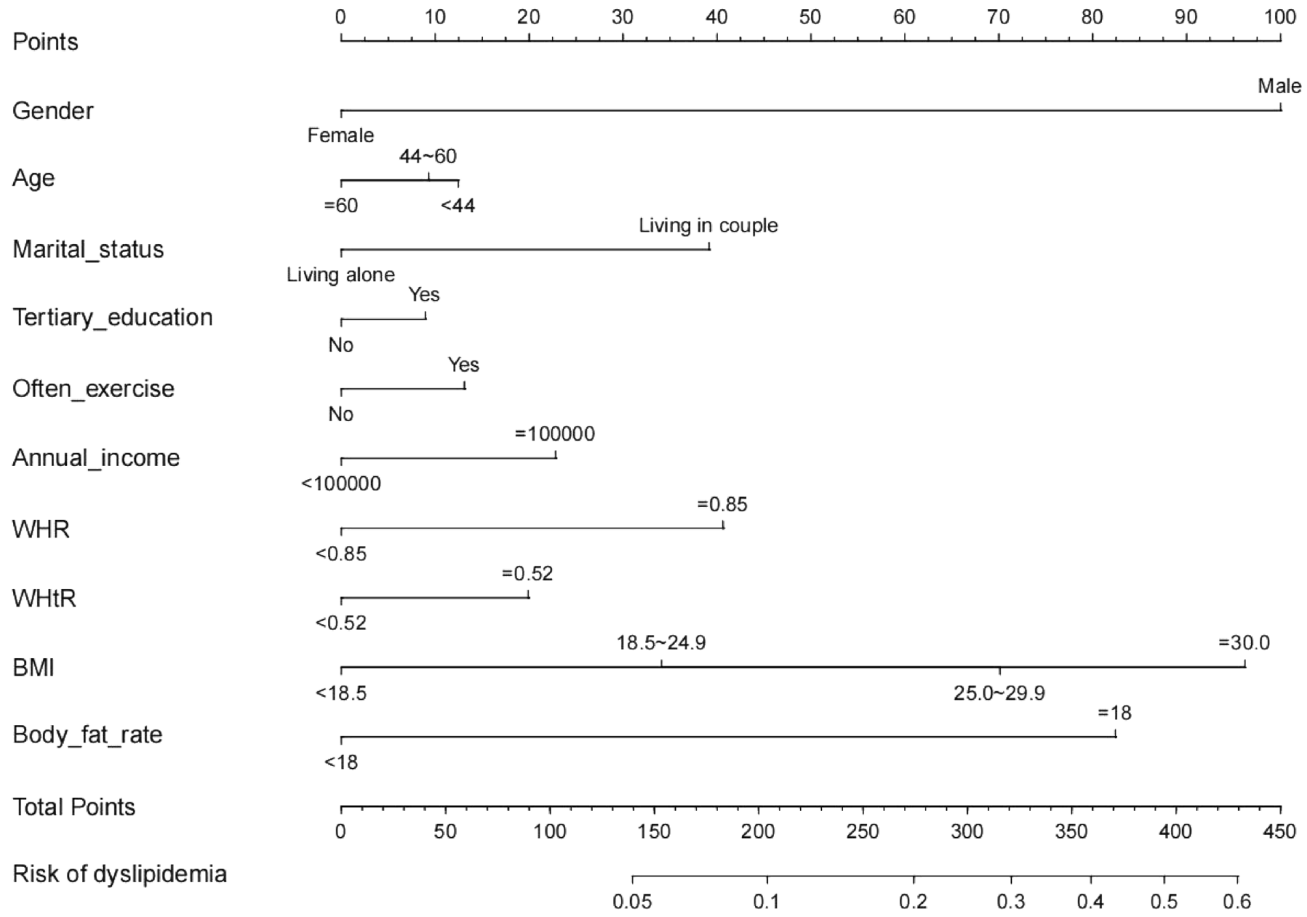
Simple-to-use nomogram for predicting dyslipidemia. The dyslipidemia nomogram was established using ten variables (Sex, Age, Marital status, Tertiary education, Annual income, WHR, WHtR, BMI, and Body fat percentage). *WHR* Waist-to-Hip Ratio, *WHtR* Waist-to-Height Ratio, *BMI* Body mass index.

Table 2 presents the results of the multivariable logistic regression analysis for sex, age, marital status, education level, physical exercise, annual income, WHR, WHtR, BMI, and body fat percentage. A model was developed by introducing the above-mentioned independent variables and presented in a nomogram (Fig. 2).

**Table 2:** Multivariate logistic regression analysis for dyslipidemia.

Variables	OR (95% CI)	*p-*value
Intercept	0.038 (0.008–0.128)	< 0.001
Sex (female vs male)	0.314 (0.209–0.470)	< 0.001
Age, years		
(44–60 vs < 44)	0.964 (0.642–1.438)	0.851
(≥ 60 vs < 44)	0.865 (0.469–1.530)	0.632
Marital status (living in couple vs living alone)	1.519 (0.978–2.412)	< 0.05
Education level (tertiary education vs no)	1.152 (0.778–1.719)	0.602
Physical exercise	1.113 (0.766–1.631)	0.428
(None vs ≤ 2 episodes/week)	0.769 (0.542–1.383)	0.151
(≥ 3 episodes/week vs ≤ 2 episodes/week)	0.696 (0.396–1.234)	0.092
Annual income, RMB (≥ 100,000 vs < 100,000)	1.313 (0.928–1.852)	0.134
WHR (≥ 0.85 vs < 0.85)	1.578 (0.989–2.561)	0.052
WHtR (≥ 0.52 vs < 0.52)	1.228 (0.814–1.854)	0.273
BMI (kg/m^2^)		
18.5–24.9 vs < 18.5	1.474 (0.505–6.284)	0.525
25.0–29.9 vs < 18.5	2.247 (0.716–9.982)	0.214
≥ 30.0 vs < 18.5	3.043 (0.824–14.926)	0.122
Body fat percentage (≥ 18% vs < 18%)	2.598 (1.497–4.656)	< 0.001

*WHR* Waist-to-Hip Ratio, *WHtR* Waist-to-Height Ratio, *BMI* Body mass index.

### Performance of the dyslipidemia nomogram

**Figure 3:**
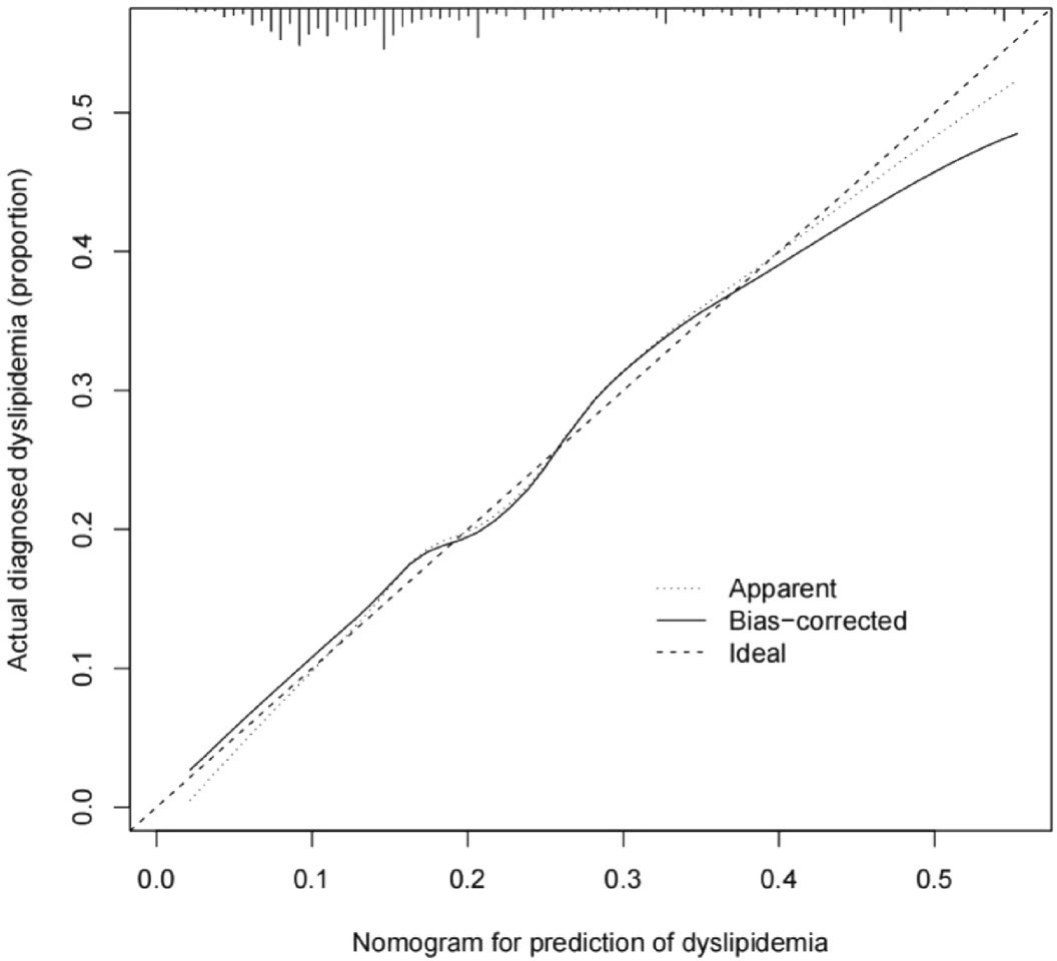
Calibration curves for the proposed nomogram in predicting dyslipidemia. x-axis, the risk of predicted dyslipidemia; y-axis, actually diagnosed dyslipidemia.

Calibration curves were plotted to assess the dyslipidemia nomogram. The calibration curve of the dyslipidemia risk nomogram for predicting dyslipidemia risk in healthy adults suggested a good performance (Fig. 3). To quantify the discrimination performance of the dyslipidemia nomogram, the C-index was calculated. The dyslipidemia nomogram was subjected to bootstrapping validation (1000 bootstraps) to calculate a relatively corrected C-index. The C-index for the prediction nomogram was 0.737 (95% CI 0.701–0.773), which suggested the model’s good discrimination ability. Good calibration and discrimination were also obtained in the internal validation with a C-index of 0.718.

### Clinical use

**Figure 4:**
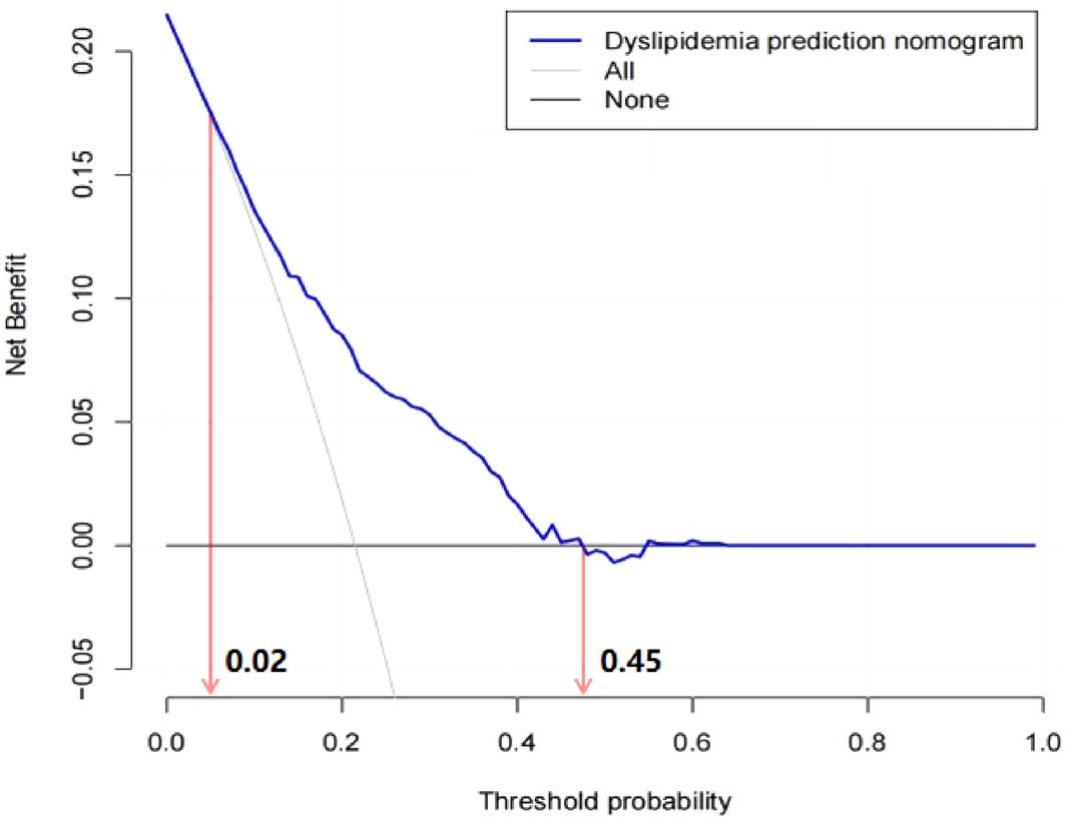
Decision curve analysis for the proposed nomogram for dyslipidemia. The decision curve showed that more benefit could be obtained from our model when the threshold probability was ranging from 0.02 to 0.45 than the intervention-all-patients scheme or the intervention-none scheme.

DCA was conducted to determine the clinical usefulness of the dyslipidemia nomogram by quantifying the net benefits at different threshold probabilities, which demonstrated that there was more benefit than either the treat-all or treat-none scheme when using the dyslipidemia nomogram at a threshold probability of 2–45% (Fig. 4).

## Conclusions

Obesity and the accompanying dyslipidemia are major risk factors for atherosclerotic cardiovascular disease (ASCVD), which could be modified by exercise^18^. In this study, we aimed to reveal the quantitative relationship between body composition index measurements of obesity and dyslipidemia. Based on our results, the self-reported healthy adults could screen for the risk of dyslipidemia themselves, and thus make an exercise plan to maintain a healthy body size and shape to reduce the risk of dyslipidemia.

The risk factors that our easy-to-use nomograms employed, including sex, age, marital status, educational level, physical exercise, annual income, WHR, WHtR, BMI, and body fat percentage, are easy to obtain. Previous studies have concluded that a C-index less than 0.7 is less accurate and that greater than or equal to 0.7 has good accuracy^19^. Internal validation in this study showed good calibration and discrimination ability of our proposed nomogram (C-index of 0.737 for the dyslipidemia nomogram and 0.718 for bootstrapping validation; both p < 0.001). The accuracy of our proposed dyslipidemia nomogram was demonstrated by the calibration curves.

Out of 1015 self-reported healthy participants in the study, the percentage of first-recorded dyslipidemia cases was 21.48% (218). In risk factor analysis, the male sex, age of 44 years or more, living as a couple, completing tertiary education, often exercising, an annual income of 100,000 RMB or more, a WHR of 0.85 or above, a WHtR of 0.52 or above, a higher BMI and a body fat percentage of 18% or above were the key individual factors that were associated with the risk of dyslipidemia.

Sex, age, obesity, and BMI were identified as common risk factors, consistent with previous studies^3,20^. Compared with the prediction model constructed by Zhang et al., the risk factors included in our model are much easier to obtain and can be used for disease screening in the general population^20^. The demographic risk factors of sex and age, which have been reported in numerous studies^7,21,22^, are non-modifiable but essential in the prediction of the risk of dyslipidemia. Living as a couple, finishing tertiary education, exercising frequently, and having an annual income of 100,000 RMB or more may indicate a better living situation, in which case it is more likely to have a high fat and calorie diet, thus contributing to increased blood lipid levels^23^. Unexpectedly, unlike previous studies, exercise tended to be a risk factor rather than a protective factor in our study. Some reasons that may explain why exercising frequently is a risk factor for dyslipidemia are as follows: reverse causality may have occurred. People who exercise frequently may have better living conditions and greater exposure to a high-fat and high-calorie diet, which may increase the proportion of individuals with this less-than-healthy diet, thereby making dyslipidemia more common. Differences in the type, intensity, and duration of exercise can have different effects on human health. Some people exercise frequently but the duration and intensity of exercise are not up to the standard, resulting in a decrease in the health benefits of exercise. Unfortunately, we did not collect data to analyze the type, intensity, and duration of exercise. The body composition indices of WHR, WHtR, BMI, and body fat percentage have been linked to dyslipidemia in numerous previous studies^24–27^. We found that the ability of BMI and body fat percentage to predict the risk of dyslipidemia was superior to that of WHR and WHtR. Consequently, we should pay more attention to maintaining a proper BMI and body fat percentage for the prevention of dyslipidemia^14^.

Dyslipidemia is a the major risk factor for the development of type 2 diabetes (T2DM), atherosclerosis, stroke, and CVDs^28–30^. In this study, the nomogram prediction tool that we developed may facilitate appropriate measures when there is more benefit than either the treat-all or treat-none schemes to control blood lipid levels and attenuate the progression of dyslipidemia-related chronic diseases. Furthermore, unlike other studies, all the risk factors included in our study could be obtained by the participants themselves, making it very easy to use.

Our study also has some limitations that warrant consideration. First, the data from a small percentage of the population in one region is not representative of all Chinese people. Second, our study was cross-sectional, and thus, we could not determine the causal relationship, and further validation in prospective studies is needed. Third, not all potential factors that could affect blood lipid levels were included in the risk factor analysis, such as eating habits, sleep disorders, etc. Finally, although the robustness of our nomogram was tested by subsequent bootstrapping validation, there was no external validation in our study to illustrate the generalizability of this model for populations in other regions and countries.

In conclusion, our proposed nomogram for dyslipidemia based on multivariate logistic regression analysis of 10 risk factors is suitable for predicting dyslipidemia risk, as evaluated by bootstrap validation. This visual and simple-to-use tool may be useful for self-reported healthy adults in self-screening the risk of dyslipidemia. It may also be helpful for dyslipidemia patients in making exercise plans to maintain a healthy body size and shape to reduce the risk of dyslipidemia.

## Data Availability

Datasets generated and/or analyzed during the current study are not publicly available due to private information about patients but are available from the corresponding author upon reasonable request.
